# miRNA in the Regulation of Skeletal Muscle Adaptation to Acute Endurance Exercise in C57Bl/6J Male Mice

**DOI:** 10.1371/journal.pone.0005610

**Published:** 2009-05-19

**Authors:** Adeel Safdar, Arkan Abadi, Mahmood Akhtar, Bart P. Hettinga, Mark A. Tarnopolsky

**Affiliations:** 1 Department of Kinesiology, McMaster University, Hamilton, Ontario, Canada; 2 Department of Pediatrics, McMaster University, Hamilton, Ontario, Canada; 3 Department of Medicine, McMaster University, Hamilton, Ontario, Canada; Universidad Europea de Madrid, Spain

## Abstract

MicroRNAs (miRNAs) are evolutionarily conserved small non-coding RNA species involved in post-transcriptional gene regulation. *In vitro* studies have identified a small number of skeletal muscle-specific miRNAs which play a crucial role in myoblast proliferation and differentiation. In skeletal muscle, an acute bout of endurance exercise results in the up-regulation of transcriptional networks that regulate mitochondrial biogenesis, glucose and fatty acid metabolism, and skeletal muscle remodelling. The purpose of this study was to assess the expressional profile of targeted miRNA species following an acute bout of endurance exercise and to determine relationships with previously established endurance exercise responsive transcriptional networks. C57Bl/6J wild-type male mice (N = 7/group) were randomly assigned to either sedentary or forced-endurance exercise (treadmill run @ 15 m/min for 90 min) group. The endurance exercise group was sacrificed three hours following a single bout of exercise. The expression of miR- 181, 1, 133, 23, and 107, all of which have been predicted to regulate transcription factors and co-activators involved in the adaptive response to exercise, was measured in *quadriceps femoris* muscle. Endurance exercise significantly increased the expression of miR-181, miR-1, and miR-107 by 37%, 40%, and 56%, respectively, and reduced miR-23 expression by 84% (P≤0.05 for all), with no change in miR-133. Importantly, decreased expression of miRNA-23, a putative negative regulator of PGC-1α was consistent with increased expression of PGC-1α mRNA and protein along with several downstream targets of PGC-1α including ALAS, CS, and cytochrome *c* mRNA. PDK4 protein content remains unaltered despite an increase in its putative negative regulator, miR-107, and PDK4 mRNA expression. mRNA expression of miRNA processing machinery (Drosha, Dicer, and DGCR8) remained unchanged. We conclude that miRNA-mediated post-transcriptional regulation is potentially involved in the complex regulatory networks that govern skeletal muscle adaptation to endurance exercise in C57Bl/6J male mice.

## Introduction

Skeletal muscle is a highly adaptable tissue that undergoes numerous metabolic and morphological adaptations in response to endurance exercise [1], [2]. Short-term and longitudinal studies have shown that endurance exercise training extends life expectancy, reduces morbidity (e.g., cardiovascular diseases, type 2 diabetes, metabolic syndrome, cancer, etc.) and physical disability in later life [3]–[5]. Endurance exercise orchestrates increases in the levels of the citric acid cycle enzymes, mitochondrial respiratory chain and the β-oxidation pathway [6]–[9]. Over the past decade, we and others have utilized global transcriptome expression technologies (e.g., oligonucleotide arrays, targeted gene expression analysis, etc.) to demonstrate that these changes are the culmination of transcriptional adaptations induced with individual, acute bouts of endurance exercise [2], [10]–[14]. Mechanisms that respond to endurance exercise stimuli are complex and involve transcriptional, post-translational, and allosteric regulation [15]–[18]. In recent years, a new level of rapid and reversible transcriptome regulation via a special class of small RNA molecules has emerged that has been speculated to fine tune gene expression and therefore may play an important role in muscle metabolic control and adaptation to exercise [19].

MicroRNAs (miRNAs) are a class of short, non-coding RNA molecules that reportedly play a central role in regulating post-transcriptional gene expression during embryonic stem cell development [20], oncogenesis [21], myogenesis [22], adipogenesis [23], fat metabolism [24], and glucose homeostasis [25]. To date, more than 500 human miRNA species have been reported, of which many are evolutionary conserved [22]. miRNAs are transcribed as long primary-miRNAs (pri-miRNA) that encode a single miRNA or a cluster of miRNA species. Genomic mapping has revealed that pri-miRNA species are encoded within non-coding genomic sequences as well as in introns or, less frequently exons, of protein-coding genes. Pri-miRNA species are processed in the nucleus by a ribonuclease III endonuclease, Drosha, and DiGeorge syndrome critical region gene 8 (DGCR8) RNA-binding protein, yielding stem-loop structures of ∼70 nucleotides, termed precursor-miRNA (pre-miRNA). These pre-miRNAs are transported to the cytoplasm by exportin-5 where they are further processed by another related ribonuclease III endonuclease, Dicer, giving rise to the mature ∼19–22 bp miRNA. The mature miRNA is incorporated into the RNA-induced silencing complex (RISC), where the miRNA strand anneals to the 3′ untranslated regions of target mRNAs to promote mRNA degradation or translational repression, but in some cases, increases its translational activity [22], [24], [26]. Estimates indicate that miRNAs may regulate up to one-third of the mammalian genome, suggesting miRNAs have a central role in regulating gene expression [27]. The versatility of miRNA-mediated gene regulation is evidenced by the finding that individual miRNAs can target hundreds of genes while individual mRNAs can be targeted by multiple miRNAs, allowing for enormous complexity and flexibility in their regulatory potential [28].

Recent studies have uncovered a cluster of muscle-specific miRNAs that are encoded by bicistronic transcripts or are nestled within introns of myosin genes [22], [26]. These miRNA species modulate diverse aspects of muscle function by acting as ‘on-off’ switches [26]. miR-1 and miR-133 are expressed in cardiac and skeletal muscle and are transcriptionally regulated by the myogenic differentiation factors MyoD, Mef2, and SRF [22]. In *Drosophila melanogaster*, deletion of miR-1 gene results in an aberrant muscle maintenance [20], [29], [30]. In contrast, miR-1 over-expression in cultured skeletal myoblasts promotes skeletal muscle differentiation [20]. Dysregulation of these myogenic miRNA species has been shown to result in numerous primary muscle disorders, including Duchenne muscular dystrophy, Becker muscular dystrophy, facioscapulohumeral muscular dystrophy, limb-girdle muscular dystrophies types 2A and 2B [31]. In addition to muscle mass maintenance, a cluster of miRNA species (miR -23, -103, -107, -278 etc.) are proposed to play an important role in regulating expression of metabolic pathways by fine-tuning gene expression patterns [24]. miRNA-mediated regulation of gene expression differs from the regulation by transcription factors in that it proceeds at a higher rate, is reversible, and allows for local changes in target mRNA and protein levels in separate cell compartments [32]. The purpose of this study is to investigate the role of miRNA in the regulation of transcriptome networks involved in mitochondrial biogenesis, glucose and fatty acid metabolism, and skeletal muscle maintenance following an acute bout of endurance exercise. Given the rapid induction of many mRNAs early following the physiological stress of acute exercise [2], we hypothesized that the miRNAs reported to be involved in regulating substrate metabolism and muscle remodelling would we temporally and directionally influenced by an acute bout of endurance exercise.

## Materials and Methods

### Animals and Exercise Program

Male C57Bl/6J mice, bred in an institutional central animal facility (McMaster University), were housed in micro-isolator cages in a temperature- and humidity- controlled room and maintained on a 12-h light-dark cycle with food and water *ad libitum*. At 4 months of age, mice (N = 7/group) were randomly assigned to either sedentary (SED) or forced-acute endurance (END) exercise bout groups ensuring that body mass was similar between groups. None of the mice had been previously subjected to a structured exercise regime. Mice in the END group were subjected to an acute bout of treadmill (Eco 3/6 treadmill; Columbus Instruments, Columbus, Ohio) running at 15 m/min for 90 min. A 5-min warm-up and cool-down at 8 m/min was also included. All of the mice in END exercise group completed the 90 min trial and were visibly exhausted (i.e., mouse will sit at the lower end of the treadmill, on the shock bar, for >5 seconds). Mice in the SED group served as controls. Three hours following the acute bout of endurance exercise, mice in both SED and END groups were euthanized by cervical dislocation and their *quadriceps* muscle extracted into RNase-free cryoviles, immediately snap frozen and stored at −80°C for subsequent analyses. The experimental protocol strictly followed guidelines put forth by Canadian Council of Animal Care and McMaster University Animal Research Ethics Board.

### Total RNA isolation

Total RNA was isolated from ∼50 mg of *quadriceps* muscle using the mirVana™ miRNA isolation kit (Ambion Inc., Austin, TX) according to the manufacturer's instructions. RNA samples were treated with TURBO DNA-*free*™ (Ambion Inc., Austin, TX) to remove DNA contamination. RNA integrity and concentration was assessed using the Agilent 2100 Bioanalyzer (Agilent Technologies, Palo Alto, CA). The average RIN (RNA integrity number) value for all samples was 8.8±0.2 (scale 1–10), ensuring a high quality of isolated RNA.

### miRNA Expression analyses

The miRNA expression was quantified in real-time using TaqMan® miRNA assays for miR -1, -23, -107, -133, and -181 according to the manufacturer's directions (Applied Biosystems Inc., Foster City, CA). Briefly, reverse transcriptase (RT) reactions were performed with miRNA-specific RT primers and 25 ng of total RNA for 30 min at 37°C followed by 10 min incubation at 95°C to inactivate the RT enzyme. End-point PCR was then performed using the RT product and microRNA-specific PCR primers for 40 cycles (two steps: 95°C for 15 s followed by 60°C for 30 s). *Rnu6* (U6 small nuclear RNA TaqMan® miRNA assay) was used as endogenous control for miRNA expression analyses.

### mRNA Expression analyses

The mRNA expression of PPARGC1 alpha (PGC-1α), citrate synthase (CS), 5-aminolevulinate synthase, (ALAS), cytochrome *c*, (cyt. *c*), pyruvate dehydrogenase kinase 4 (PDK4), Drosha, DiGeorge syndrome critical region gene 8 (DGCR8) and Dicer were quantified using 7300 Real-time PCR System (Applied Biosystems Inc., Foster City, CA) and SYBR® Green chemistry (PerfeC_T_a SYBR® Green Supermix, ROX, Quanta BioSciences, Gaithersburg, MD) as previously described [2]. First-strand cDNA synthesis from 1 µg of total RNA was performed with random primers using a high capacity cDNA reverse transcription kit (Applied Biosystems Inc., Foster City, CA) according to manufacturer's directions. Forward and reverse primers (Table 1) for the aforementioned genes were designed based on sequences available in GenBank (http://www.ncbi.nlm.nih.gov/entrez/query.fcgi) using the MIT Primer 3 designer software (http://wi.mit.edu/cgi-bin/primer3/primer3_www.cgi), and were confirmed for specificity using the basic local alignment search tool (www.ncbi.nlm.nih.gov/BLAST/). β-2 microglobulin was used as a control house-keeping gene. All samples were run in duplicate simultaneously with negative control which contained no cDNA. Melting point dissociation curves generated by the instrument was used to confirm the specificity of the amplified product.

**Table 1:** Real-time PCR primer sequences.

Gene	Accession Number	Forward primer (5′→3′)	Reverse primer (5′→3′)
PGC-1α	NM_008904	ttccaccaagagcaagtat	cgctgtcccatgaggtatt
PDK4	NM_013743	aagatgctctgcgaccagtat	gaaggtgtgaaggaacgtaca
ALAS	NM_009653	aagggcactggtcggttta	ctgagggactcgggataaga
CS	NM_026444	gcatgaagggacttgtgta	tctggcactcagggatact
cyt. *c*	NM_007808	cacgctttacccttcgttct	ctcatttccctgccattctcta
β-2 microglobulin	NM_009735	ggtctttctggtgcttgtct	tatgttcggcttcccattct

### *Quadriceps* Muscle homogenization

Total protein was extracted from frozen skeletal muscle samples as detailed previously [13]. Briefly, ∼30 mg of *quadriceps* muscle was homogenized on ice in a 2 mL Wheaton glass homogenizer (Fisher Scientific, Ottawa, ON) with 25 volumes of phosphate homogenization buffer [50 mM KPi, 5 mM EDTA, 0.5 mM DTT, 1.15% KCl supplemented with protease inhibitor cocktail (Complete Mini, ETDA-free, Roche Applied Science, Manheim, Germany) and phosphatase inhibitor cocktail (PhosSTOP, Roche Applied Science, Manheim, Germany). The lysate was centrifuged at 700 *g* for 15 min at 4°C to separate cellular debris. The supernatant was aliquoted, snap frozen in liquid nitrogen and stored at −80°C for further analysis. The BCA protein assay (Pierce, Thermo Fisher Scientific, Nepean, ON) was used to quantify the total protein content of samples.

### Immunoblotting

Proteins were resolved on 10% SDS-PAGE gels and were transferred onto Hybond® ECL nitrocellulose membranes (Amersham, Piscataway, NJ) followed by blocking with 5% milk in TBST overnight at 4°C. Immunoblotting was carried out using rabbit monoclonal PGC-1α (3G6) antibody (1∶1000 dilution; Cell Signaling Technology, Danvers, MA) and PDK4 antibody (1∶2500 dilution; Abcam, Inc., Cambridge, MA). Membranes were then incubated with anti-rabbit horseradish peroxidase-linked secondary antibody (1∶5000 dilution, Bio-Rad Laboratories, Burlington, ON.) and were visualized by enhanced chemiluminescence detection reagent (Amersham, Piscataway, NJ). Relative intensities of the protein bands were digitally quantified by using NIH ImageJ, version 1.37, analysis software (Scion Image, NIH). Actin (BD Biosciences, Mississauga, ON) was used as a house-keeping protein.

### Statistics

Data were tested for normality using the Kalmagarov-Smirnov test (GraphPad Prism 4.0, La Jolla, CA) and were found to be normally distributed. Unpaired Student *t*-tests were used to test for differences between groups. Linearized 2^−ΔCt^ (fold-change) measurements were used for miRNA and mRNA expression, and arbitrary units for PGC-1α and PDK4 protein content (Statistica 5.0, Statsoft, Tulsa, OK). Linear regression was carried out to define correlation between PGC-1α content and miR-23 expression. Statistical significance was established at P≤0.05. Data are presented as the means±standard deviation (SD).

## Results

### An acute bout of endurance exercise results in the down-regulation of miR-23 and increases in cellular PGC-1α protein content along with several of its downstream mitochondrial targets

**Figure 1:**
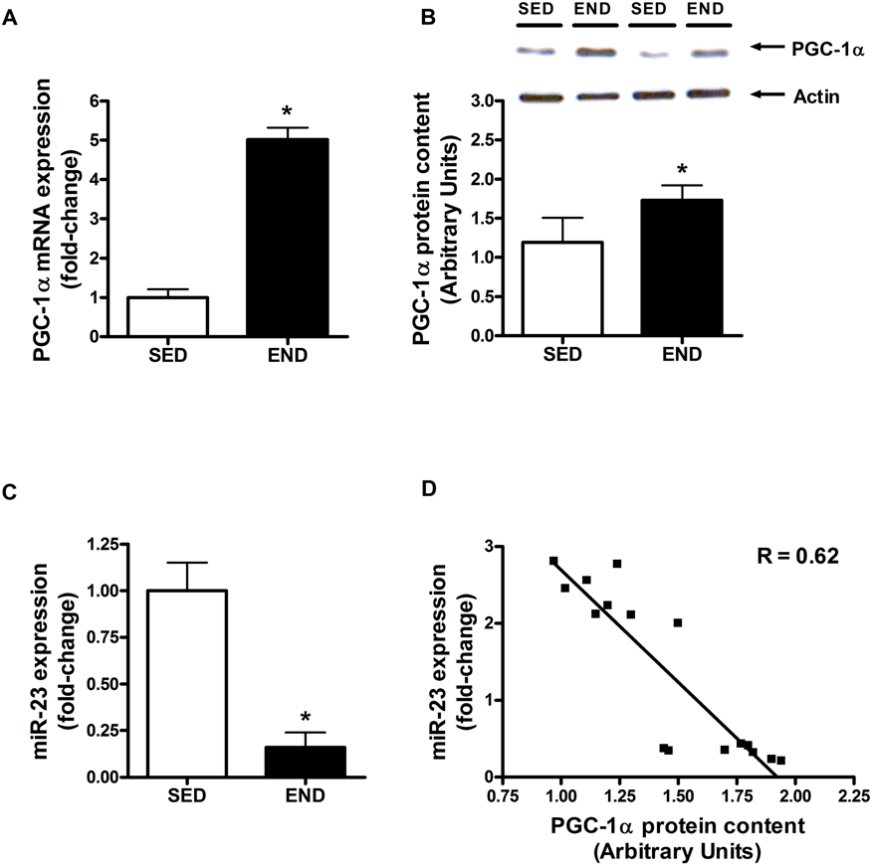
PGC-1α content and miR-23 expression following exercise. PGC-1α (A) mRNA expression and (B) protein content, and (C) miR-23 expression in the *quadriceps* of C57Bl/6J mice (N = 7/group) 3-hour following an acute bout of END exercise vs. SED group. (D) PGC-1α protein content negatively correlates (R = 0.62) with miR-23 content. PGC-1α mRNA expression, protein content and miR-23 expression are normalized to β-2 microglobulin, actin and *Rnu6*, respectively. Asterisks denote significant changes (P≤0.05).

**Figure 2:**
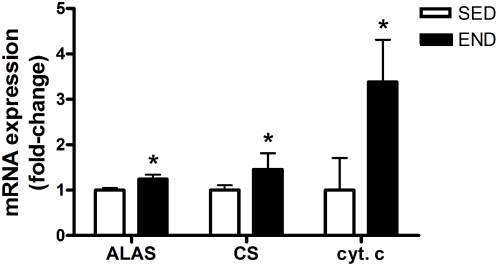
Transcription of PGC-1α target genes following exercise. Activation of ALAS, CS and cyt. *c* mRNA expression (fold-change) in the *quadriceps* of C57Bl/6J mice (N = 7/group) 3-hour following an acute bout of END exercise vs. SED group. ALAS, CS and cyt. *c* mRNA expression are normalized to β-2 microglobulin. Asterisks denote significant changes (P≤0.05).

PGC-1α mRNA expression (3.0-fold vs. SED group; P<0.001) and protein content (45% vs. SED mice; P = 0.018) were increased in *quadriceps* three hours post-exercise (Figure 1A and 1B). miR-23 expression, a putative regulator of PGC-1α mRNA translation [24], was significantly decreased (84% END vs. SED group; P<0.001) at this time point (Figure 1C). The increase in PGC-1α protein content was significantly negatively correlated with decreased expression of miR-23 (R = 0.62; P = 0.032; Figure 1D). To further confirm the increase in PGC-1α content, we assessed the mRNA expression of mitochondrial biomarkers (ALAS, CS and cyt. *c*) that are co-activated by PGC-1α. ALAS, CS and cyt. *c* mRNA expression was significantly up-regulated by 1.2, 1.5 and 3.5 fold, respectively, in response to an acute bout of END exercise (P<0.04; Figure 2).

### An acute bout of endurance exercise results in the up-regulation of miR-107 and PDK4 mRNA expression

**Figure 3:**
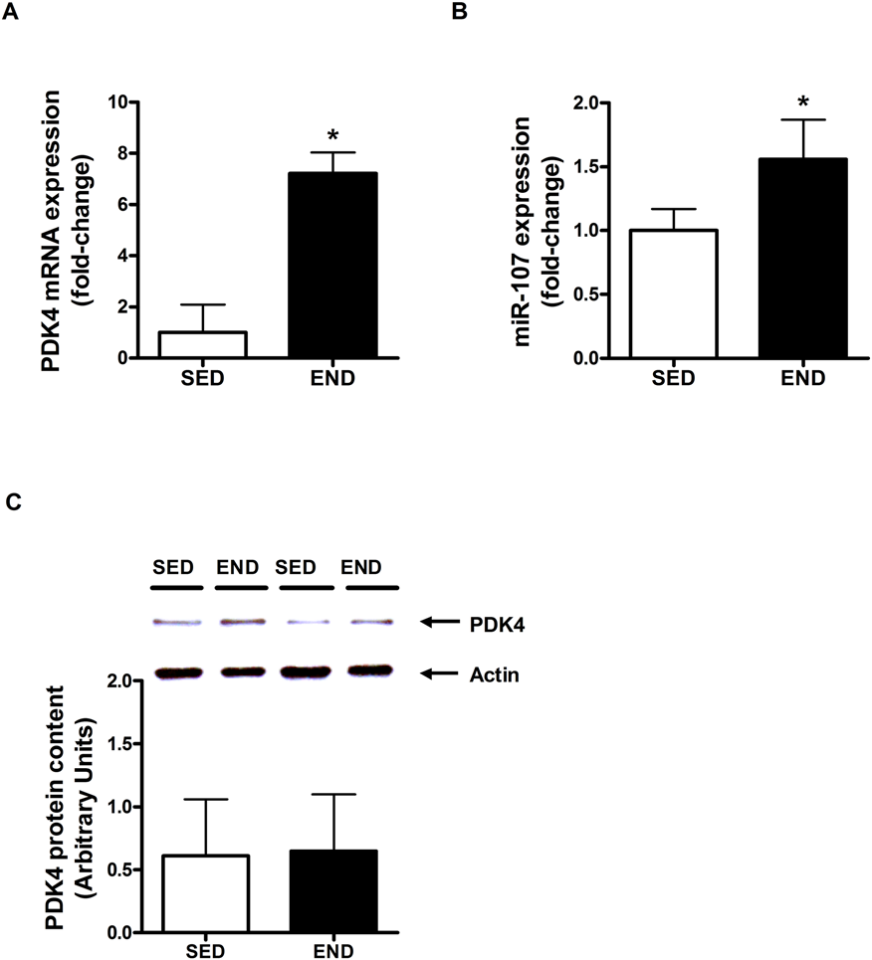
PDK4 content and miR-107 expression following exercise. (A) PDK4 mRNA expression and (B) protein content, and (C) miR-107 expression in the *quadriceps* of C57Bl/6J mice (N = 7/group) 3-hour following an acute bout of END exercise vs. SED group. PDK4 mRNA expression, protein content and miR-107 expression are normalized to β-2 microglobulin, actin and *Rnu6*, respectively. Asterisks denote significant changes (P≤0.05).

miR-107 was predicted to regulate the expression of PDK4 [24], an important component of the cellular response to endurance exercise [2]. Both PDK4 mRNA (7.2-fold END vs. SED group; P<0.001) and miR-107 expression (56% vs. SED group; P<0.001) were increased in the *quadriceps* muscle of mice three hours following an acute bout of END exercise (Figure 3 A and C). However, PDK4 protein content remained unchanged (Figure 3B).

### An acute bout of endurance exercise results in the up-regulation of miR-1 and miR-181

**Figure 4:**
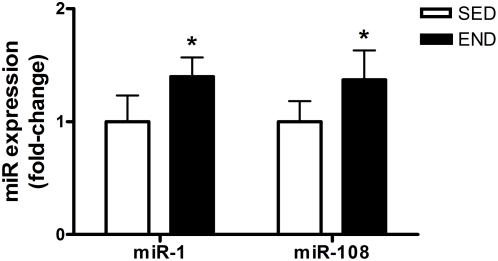
miR-1 and miR-181 expression following exercise. miR-1 and miR-181 expression in the *quadriceps* of C57Bl/6J mice (N = 7/group) 3-hour following an acute bout of END exercise vs. SED group. miR-1 and miR-181 expression are normalized to *Rnu6*. Asterisks denote significant changes (P≤0.05).

miR-1 and miR-181 are thought to play an important role in muscle differentiation and development as positive regulators of skeletal muscle remodeling and maintenance [26]. Both miR-1 and miR-181 expression, were increased in *quadriceps* by 40% and 37% (END vs. SED; P<0.05), respectively, three hours following an acute bout of END exercise (Figure 4). miR-133 expression was not altered in response to END exercise.

### An acute bout of endurance exercise does not alter mRNA expression of nuclear and cytoplasmic miRNA processing complexes

mRNA expression of both nuclear (Drosha and DGCR8) and cytoplasmic (Dicer) miRNA processing complexes remained unchanged in *quadriceps* muscle three hours following an acute bout of END exercise (data not shown).

## Discussion

In this study, we found that down-regulation of miR-23 is associated with a significant increase in PGC-1α mRNA expression and protein content in *quadriceps* of C57Bl/6J male mice three hours following an acute bout of endurance exercise. This increase in PGC-1α protein coincided with the up-regulation of mRNA expression of ALAS, CS and cytochrome *c*, all of which are co-activated by PGC-1α. In addition, we report a significant induction in miR- 107, 1 and 181 in the skeletal muscle of male mice subjected to endurance exercise. mRNA expression of miRNA nuclear and cytoplasmic processing machinery remain unaltered with endurance exercise. The important novel information emerging from the current study is that the physiological stress of a single bout of endurance exercise is sufficient to alter miRNA abundance for species involved in regulating skeletal muscle metabolism and maintenance.

In skeletal muscle, an acute bout of endurance exercise up-regulates transcriptional networks involved in mitochondrial biogenesis, lipid and carbohydrate metabolism, oxidative stress management, interferon signaling, electrolyte transport across membranes, and extracellular matrix remodeling [2]. The cumulative effect of the activation of these pathways is two-tier; to achieve homeostatic recovery and to induce adaptations to successive bouts of exercise [2]. The general consensus is that skeletal muscle gene expression in response to endurance exercise is an early event that initiates many aspects of physiological adaptation, which not only improves exercise performance, but also mediates some of the protective effects of exercise against obesity and associated metabolic disorders [3]. Studies have shown that the myriad of transcriptome networks induced by endurance exercise are under the strict transcriptional and translational control of complex signal transduction pathways, post-translational modifications and allosteric regulation [12], [16], [18]. First described in *Caenorhabditis elegans* as small temporal RNA molecules required for proper development, miRNAs are now recognized as a new class of *trans*-factors that regulate gene expression, which until now have been the exclusive domain of proteins [28], [33]. Their ability to induce rapid and reversible changes in target mRNA and protein content makes them an excellent candidate to orchestrate cellular homeostasis and adaptive responses in the early recovery hours following acute endurance exercise [19], [32].

Endurance exercise is a potent inducer of mitochondrial biogenesis in skeletal muscle [2], [34]. During mitochondrial biogenesis nuclear- and mitochondrial DNA- encoded gene expression must be co-ordinated [35]. The transcriptional co-activator PGC-1α has been termed a master regulator of this response owing to its ability to co-activate several nuclear transcription factors and increase the expression of mitochondrial transcription factor A [35]–[37]. Over-expression of PGC-1α in muscle cells increases mitochondrial content and oxidative capacity [38]. Similarly, transgenic over-expression of PGC-1α increases the mitochondrial content of mouse skeletal muscle and conversion of low oxidative white muscle fibres to high oxidative red muscle fibres [39]. We observed a significant increase in both PGC-1α mRNA expression and protein content three hours following an acute bout of endurance exercise (Figure 1A and 1B). Wright et al. (2007) reported similar increases in PGC-1α protein content three hours following an exhaustive bout of endurance exercise in rats [40]. The induction of PGC-1α may be partly mediated by ATF-2 and MEF2 which were recently shown to act on the PGC-1α promoter and induce PGC-1α expression in response to exercise stimulus [40]. However the significant induction in protein content of PGC-1α after three hours of exercise appears too rapid to be just driven by transcription factors. Recently, Wilfred et al. (2007) mined four public databases (MiRANDA: 2005 build, MiRANDA: 2006 build, PicTAR, and TargetScan 2006 build) to forecast putative miRNA species that regulate mRNA species involved in the regulation metabolic pathways [24]. They identified miR-23 as a putative regulator of PGC-1α protein content. Indeed we observed a significant reduction in miR-23 transcript following acute endurance exercise (Figure 1C) which was significantly correlated with increases in PGC-1α protein content (Figure 1D). The association between a reduction in miR-23 expression and an increase in PGC-1α protein content indicates that miR-23 may negatively regulate PGC-1α protein expression. During the recovery period following exercise, the decrease in miR-23 may be permissive for an increase in PGC-1α protein, possibly via increased translation or stability of PGC-1α mRNA. The increased PGC-1α protein then drives an increase in mitochondrial biogenesis, as evidenced by an increase in ALAS, CS, cytochrome *c* mRNA expression (Figure 2). In addition to exercise response, PGC-1α has been conclusively implicated in ameliorating disuse-induced muscle atrophy [41], Duchenne muscular dystrophy [42], and statin-mediated muscle wasting [43] in animal models. We speculate that dysregulation of miR-23 expression may be partly responsible for the etiology of these pathologies, and modulation of miR-23 could be a future therapeutic target for conditions where physical activity is not medically feasible.

In addition to mitochondrial biogenesis, endurance exercise also induces β-oxidation pathways to spare glucose during the recovery period following acute exercise [2], [6], [7]. As previously reported by us [2] and other groups [44]–[46], we observed a significant increase in skeletal muscle PDK4 mRNA content three hours following acute exercise (Figure 3A). PDK4 is a member of a family of protein kinases that phosphorylate and inactivate the E1α subunit of pyruvate dehydrogenase complex, thus preventing the entry of glycolytic products into the mitochondria for oxidation [47], [48]. In skeletal muscle, PDK4 is induced in response to fasting, high-fat feeding, and endurance exercise, all of which represent metabolic states where there is a deficit in whole body glucose availability, and thus a transition from carbohydrate to non-esterified fatty acid metabolism is warranted [44], [49], [50]. It is thought that persistent elevation of PDK4 expression during recovery from exercise ensures that glucose entering the cell is preferentially used for muscle glycogen resynthesis [44]. miR-103 and miR-107, which exist in vertebrate genomes within introns of the pantothenate kinase (PANK) genes, are predicted by Wilfred et al. (2007) to affect multiple mRNA targets in fatty acid synthesis and utilization [24]. A peroxisome proliferator-activated receptor-alpha (PPAR-α) targeted promoter is recently described up-stream of the PANK1 gene [51]. Since PPAR-α receptors are stimulated by increased intracellular lipids/fatty acids; the function of PPAR-α is thought to involve decreasing intracellular fatty acid stores [52], [53]. Wilfred et al. (2007) suggested that miR-103/7 cooperate with the PANK proteins and PPAR-α to decrease fatty acid synthesis and increase the activity of the pyruvate dehydrogenase complex (by inhibiting PDK4, PISD, and PDPR) [24]. We observed an increase in miR-107 content (Figure 3C) as well as PDK4 mRNA (Figure 3A) following acute exercise however no significant change in PDK4 protein content was detectible (Figure 3B). We believe that the increase in PDK4 mRNA after a single bout of endurance exercise may occur in preparation for future bouts of similar exercise, i.e., the potential need to metabolize more fat. These early modes of adaptation are mediated largely at the transcriptional level [2]. It is thought that “pulses” of elevated mRNA expression after individual exercise bouts within a training program will lead to long-term increases in protein abundance, which will culminate in physiologic adaptation to exercise [2]. We believe that one mode of restricting early transcriptional responses from occurring at the protein level, until ‘sufficient’ exercise bouts have been performed, is via inhibition of the translation mRNA species by specific miRNA species. Hence, even though we observe an increase in PDK4 mRNA expression (Figure 3A), post-transcriptional inhibition by miR-107 prevents translation of PDK4 mRNA thus preventing an increase in PDK4 protein content (Figure 3B). Recently Wang and colleagues (2008) have reported that a decrease in miR-107 expression may accelerate progression of Alzheimer disease via dysregulation of β-Site amyloid precursor protein-cleaving enzyme 1 [54]. Exercise training has been shown to decrease frailty and improve physical health in patients with Alzheimer disease, and is associated with delay in onset of dementia and Alzheimer disease [55], [56]. Here we have observed an up-regulation of miR-107 with an acute bout of endurance exercise. Taken together, these data suggest that in addition to regulation PDK4 expression, miR-107 modulation with endurance exercise may have therapeutic consequences.

Endurance exercise training maintains skeletal muscle mass in an activity-dependent manner, therefore each bout of endurance exercise induces myogenic factors that promote skeletal muscle remodelling [57], [58]. miR- 1 and 133 are highly expressed in skeletal muscle and are transcribed from a common polycistronic gene during development [22], [26]. Chen et al. (2006) have demonstrated that these miRNA species have opposing roles in modulating skeletal muscle proliferation and differentiation in cultured myoblasts *in vitro* and in *Xenopus laevis* embryos [59]. miR-1 promotes myogenesis by targeting histone deacetylase 4, a transcriptional repressor of muscle gene expression [59]. On the other hand, miR-133 enhances myoblast proliferation by repressing the serum response factor [59]. In addition, miR-181 was found to be strongly up-regulated in regenerating muscle from an *in vivo* mouse model of muscle injury [60]. miR-181 is thought to function partly through inhibition of Hox-A11 expression [60]. Hox-A11 is a known repressor of MyoD which in turn is required for new muscle growth [60]. We have observed a significant increase in skeletal muscle miR- 1 and 181 content three hours following acute exercise (Figure 4). miR-133 content remained unchanged in both sedentary and forced-endurance exercise groups. We hypothesize that increases in miR- 1 and 181 partly repress factors that negatively regulate myogenic expression, and thus promote skeletal muscle remodelling following a damaging endurance bout. Recently, McCarthy and Esser (2007) indicated that miR- 1 and 133a were down-regulated after 7 days of functional overload in mice [61]. The authors concluded that decreases in miR- 1 and 133 are needed to promote expression of genes involved in skeletal muscle hypertrophy, which is characteristic of resistance training [61]. This is consistent with our contention that increased miR- 1 and 133 are involved in maintenance of skeletal muscle mass, which is characteristic of endurance training. Lastly, a recent study has shown that miR-1 levels are significantly increased in rat cardiomyocytes in response to oxidative stress [62]. We have previously shown a coordinated induction of the metallothionine gene family, involved in free radical management, three hours following an acute bout of endurance exercise [2]. Thus increased miR-1 in skeletal muscle of exercised mice may represent an adaptive response to oxidative stress imposed by acute exercise. We speculate that miR-1 may also play a role in inducing antioxidant response in skeletal muscle.

The findings of this study highlight the complexity of coordinated gene expression and transcriptional circuits that control skeletal muscle gene expression during recovery period following an acute bout of endurance exercise. It is important to mention that our experiment is limited to measurement of a few select miRNA species in male mice. It is likely that other miRNA species (as well as additional miRNA targets) play a role in regulating homeostatic recovery and adaptive responses in skeletal muscle following acute exercise, and that there may be sex differences in miRNA regulation in response to exercise stimulus. Our results support a potential role for miRNA in regulating the transcriptional responses to exercise and support further miRNA profiling to map out the global role of miRNA-mediated transcriptome regulation in exercise. In addition, further work is needed to elucidate potential upstream regulators of miRNA induction in response to acute exercise, i.e., calcium/calmodulin signalling, localized hypoxia, changes in cellular energy changer (AMP/ATP and NAD^+^/NADH ratio), and mechanical stretch. Since endurance exercise is suggested to have therapeutic potential against obesity, type II diabetes, metabolic syndrome and associated co-morbidities [3]–[5], it is intriguing to speculate that maintenance of the miRNA signalling networks via endurance exercise training may combat these pathologies.
